# Left ventricular systolic longitudinal strain in mechanically ventilated patients in the intensive care unit: assessment of global and chamber reproducibility

**DOI:** 10.1186/s40635-025-00770-8

**Published:** 2025-06-17

**Authors:** Matías Pécora, Piero Pastorini, Roberto Farolini, Gastón Burghi, F. Javier Hurtado

**Affiliations:** 1 Unidad Académica de Fisiopatología, Hospital de Clínicas, Facultad de Medicina, Universidad de la República, Montevideo, Uruguay; 2 Laboratorio de Exploración Funcional Respiratoria, Centro de Tratamiento Intensivo, Hospital de Clínicas, Facultad de Medicina, Universidad de la República, Montevideo, Uruguay; 3 Unidad de Cuidados Intensivos – Hospital Español, Administración de los Servicios de Salud del Estado (ASSE), Montevideo, Uruguay

**Keywords:** Left ventricular systolic longitudinal strain, Myocardial failure, Mechanical ventilation, Left ventricular failure, Left ventricular ejection fraction

## Abstract

**Introduction:**

In the intensive care unit (ICU), left ventricular systolic function is traditionally assessed by measuring the left ventricular ejection fraction (LVEF). Recently, left ventricular global systolic longitudinal strain (SL-S) has emerged as a more sensitive marker of myocardial function in this setting. However, obtaining high-quality echocardiographic images remains a significant challenge, particularly in patients undergoing invasive mechanical ventilation (IMV), and data on the feasibility and reproducibility of these measurements in critically ill patients are limited.

**Objective:**

To assess the feasibility and reproducibility (both global and per chamber) of SL-S and LVEF (both manual and automatic) in ICU patients under IMV.

**Materials and methods:**

Thirty ICU patients receiving IMV were randomly selected. The feasibility and reproducibility of SL-S (global and per chamber) and LVEF were assessed using both manual and automatic methods. The analysis was performed using the intraclass correlation coefficient (ICC) with its 95% confidence interval (CI), and Bland–Altman analysis (BA), which reported the mean difference and limits of agreement (lower–upper limits of agreement).

**Results:**

SL-S was feasible in 70% of patients and demonstrated excellent intra- and interobserver reproducibility for both manual and automatic methods. Intraobserver reproducibility for automatic SL-S: ICC 0.97 (CI: 0.94–0.99), BA 0.26 (−1.89 to 2.40) and interobserver reproducibility: ICC 0.96 (CI: 0.92–0.98), and BA 0.53 (−2.41 to 3.47). The reproducibility of manual SL-S was comparable to automatic measurements. Additionally, the reproducibility per chamber was excellent. LVEF was feasible in 80% of patients. Manual LVEF (Simpson’s biplane) reproducibility demonstrated good reproducibility: intraobserver ICC: 0.82 (CI: 0.48–0.93), BA −5.00 (−19.70 to 9.70); interobserver ICC 0.78 (CI: 0.55–0.91), BA 7.50 (−5.40 to 20.40). Automatic LVEF (auto-LVEF) demonstrated excellent reproducibility: intraobserver ICC: 0.94 (CI: 0.86–0.98), BA −0.95 (−10.02 to 8.13); and interobserver ICC: 0.94 (CI: 0.87–0.97), BA 1.75 (−6.38 to 10.33).

**Conclusion:**

SL-S (global and per chamber) and auto-LVEF were feasible and showed excellent reproducibility. LVEF demonstrated the highest feasibility, while SL-S exhibited the greatest reproducibility. These parameters may represent a useful tool in the evaluation of LV function in ICU patients under IMV.

**Supplementary Information:**

The online version contains supplementary material available at 10.1186/s40635-025-00770-8.

## Keypoints

- Question: Is SL-S feasible and reproducible in the ICU compared to LVEF in patients under invasive mechanical ventilation?
- Findings: SL-S demonstrated 70% feasibility, exhibited excellent reproducibility for both global and chamber assessments and better reproducibility than LVEF, which displayed 80% feasibility and excellent reproducibility by auto-LVEF. Simpson’s biplane LVEF showed good reproducibility.
- Significance: SL-S and auto-LVEF proved to be feasible and reproducible parameters for assessing LV systolic function in patients undergoing invasive mechanical ventilation.

**Supplementary Information:**

The online version contains supplementary material available at 10.1186/s40635-025-00770-8.

## Introduction

Two-dimensional echocardiography utilizing speckle tracking enables the quantification of myocardial velocity and deformation, offering a novel perspective on assessing systolic and diastolic function through muscle fiber performance. Speckle tracking employs a semi-automated algorithm to track myocardial segments (speckles) during the cardiac cycle, comparing the final length to its resting length at end-diastole [1]. During systole, the distance between the speckles decreases, resulting in negative strain values. More negative values indicate better systolic function. The most widely used strain parameter with substantial evidence is left ventricular global systolic longitudinal strain (SL-S) [2–5]. SL-S is highly applicable in cardiology, particularly in areas such as cardio-oncology and in conditions like amyloidosis, sarcoidosis, valvular cardiomyopathies, pulmonary hypertension, and heart failure with preserved LVEF [1, 6]. SL-S represents a more sensitive parameter for detecting subtle myocardial dysfunctions and provides additional prognostic information that is not evident through conventional echocardiographic techniques, including left ventricular ejection fraction (LVEF), which is the most used and accepted parameter of LV systolic function [1, 7, 8]. Furthermore, SL-S demonstrates high feasibility and excellent reproducibility, with the latter being superior to that of LVEF by modified Simpson’s biplane [7–11].

In the ICU setting, echocardiography is a fundamental and widely accessible tool for assessing cardiac function in various conditions or scenarios. LV systolic function is typically assessed by LVEF measurement by modified Simpson’s biplane method [12, 13]. However, LVEF presents various disadvantages. First, it is a volumetric measure; it can be normal despite left ventricular dysfunction. Second, it is strongly influenced by loading conditions, which are often altered in critically ill patients (e.g., peripheral vasodilation, vasopressor use, hypovolemia), thereby limiting its utility for evaluating intrinsic myocardial contractility [12, 14, 15]. The role of LVEF is controversial in sepsis/septic shock or suspected septic cardiomyopathy, as it has not demonstrated convincing prognostic value [16–20]. These results may arise from dependence on loading conditions, geometric assumptions, inability to detect passive or active displacement of the endocardium, and the reproducibility of measurements in critically ill patients [20]. Furthermore, the lack of validated automated software for measurements may restrict the reproducibility, widespread applicability, and clinical utility of LVEF within the ICU context.

In the ICU, SL-S has emerged as a valuable tool, particularly for diagnosing and prognostic evaluation in septic cardiomyopathy [21–25]. Evidence suggests that SL-S can detect subtle myocardial dysfunction even before reductions in LVEF become apparent [15, 26]. Despite its potential, SL-S remains partially influenced by loading conditions [15, 27]. A critical aspect of SL-S is its feasibility and reproducibility in the ICU setting prior to its applicability and clinical usefulness. This is particularly relevant in patients under invasive mechanical ventilation (IMV), with limited cooperation, making the acquisition of high-quality images technically challenging. Existing literature on ICU feasibility and reproducibility does not solely focus on those under IMV or does not provide specificity on this aspect. [17, 21, 28–32]. This is relevant as IMV is widely acknowledged in the literature [24].

Moreover, evidence on the performance of fully automated software for measuring LVEF in critically ill patients remains limited [33]. Therefore, assessing the reproducibility of both SL-S and LVEF in the ICU is crucial to establish their clinical utility, especially given the variability reported across studies, which directly impacts the strength of recommendations for their use [34]. The study aims to evaluate and compare the reliability of manual and automated SL-S and LVEF measurements in ICU patients undergoing IMV.

## Methods

The methodology adhered to the recommendations of the “Preferred Reporting Items for Critical Care Echocardiography Studies” (PRICES) consensus [34].

### Study population

This single-center analytical, cross-sectional observational study was conducted at the Intensive Care Medicine Unit of the University Hospital, Hospital de Clínicas, School of Medicine. The research was conducted in accordance with the Declaration of Helsinki—Ethical Principles for Research on Human Beings. The study was presented and approved by the Research Ethics Committee of the Hospital de Clínicas (Resolution N°109-22). Written informed consent was obtained from all legal representatives.

In line with our ICU clinical protocols, all the patients on invasive mechanical ventilation receive hemodynamic evaluation through echocardiography. This ICU is equipped with 23 beds. Thirty patients were randomly selected using a random number generator app. The selection occurred over successive days, regardless of the time of evolution in the ICU. Each patient was included only once. The patients were randomly selected to minimize selection bias, particularly concerning the inclusion of individuals with body types that may favor optimal echocardiographic windows (e.g., lean subjects). (Supplementary material; Appendix 1). Inclusion criteria were age ≥ 18 years, informed consent, and undergoing IMV. Exclusion criteria were the presence of atrial fibrillation and immediate postoperative cardiac surgery.

Clinical data (ICU admission criteria, mean arterial pressure, vasopressor use, heart rate, tidal volume, positive pressure at end expiration) and comorbidities (obesity, chronic obstructive pulmonary disease, hypertension, diabetes, chronic heart failure, acute myocardial infarction, chronic kidney disease) were obtained through a comprehensive review of the patient’s medical records at the time of echocardiography. ICU length of stay and ICU mortality were collected prospectively. Disease severity was evaluated using the Simplified Acute Physiology Score III (SAPS 3) at ICU admission [35].

### Conventional transthoracic echocardiography

Vivid ultrasound from GE Healthcare^®^ using a 3–5 MHz transducer was employed.

Patients were in the supine position. Transthoracic echocardiography was conducted according to established guidelines [36, 37].

Quantification of the LV linear thickness. LV volumes and LVEF were calculated using the modified Simpson’s biplane method. Mitral inflow measurement utilized pulsed-wave Doppler to assess peak E wave. Tissue Doppler imaging was employed to measure early diastolic velocities (e′) at both the septal (e′ septal) and lateral (e′ lateral) mitral annulus, and the average of these two values (e′ average) was calculated. The E/e′ ratio was derived using the pulsed Doppler E wave and the average e′ (E/e′ average). Left atrial volume was measured at the four-chamber view. Basal right ventricular diameter was assessed at the four-chamber view. Right ventricular systolic function was evaluated using tricuspid annular plane systolic excursion.

### Speckle tracking imaging

Speckle tracking analysis was performed using GE^®^ Echo-Pac version 110.

LV images were obtained from the apical view of the two, three, and four chambers, thereby ensuring the exclusion of apical foreshortening and the provision of adequate endocardial border definition. Images with a frame rate of 40–80 frames per second were selected for analysis [38]. Five cardiac cycles triggered by electrocardiogram were recorded, and the clearest cardiac cycle was selected for strain analysis. End diastole was defined as the closure of the mitral valve identified on transmitral pulsed Doppler. End systole was defined by the closure click detected on the spectral tracing of the LV outflow tract pulsed Doppler [39, 40]. Peak systolic strain was recorded for each chamber and globally before or at the closure of the aortic valve [41]. SL-S was expressed as a percentage change (%) in negative values, indicating myocardial shortening during systole. It corresponds to the global longitudinal peak strain, calculated as the average from the 17 segments of the LV, derived from apical two, three, and four chamber views. SL-S analysis was performed offline on the workstation using two modes: “Automated Functional Imaging” (AFI) and manual “Q analysis”.

AFI: The operator defines three points (two at the mitral annulus and one at the apex) for each apical view. The software then displays the region of interest (ROI) on the myocardial wall. When necessary, manual adjustments were made to the automatic endocardial recognition to ensure that the algorithm correctly followed the myocardial wall.

The software divides each apical view into six equidistant segments and provides the peak SL-S value for each segment and each view, as well as the average from the three apical views (Supplementary material; Appendix 2).

Q analysis: The ventricular endocardial edges are manually traced for the three apical views. The ROI width is defined based on ventricular thickness. The software divides the cavity into six equidistant segments and provides SL-S curves, peak values for each segment, each view, and the average from the three apical views (supplementary material; Appendix 3).

Exclusion of two or more myocardial segments per apical view due to poor tracking quality (AFI or Q analysis) was considered a criterion for non-feasibility of strain analysis [38, 41].

### LVEF assessment

LVEF was assessed using two methods:

Manual method: modified Simpson’s biplane method (modified Simpson’s rule), currently the recommended approach [37].

Automatic method: “AutoEF” by GE^®^ (auto-LVEF), although not included in the latest guidelines, is increasingly used. This method defines three points in the apical view of the two and four chambers (two at the mitral annulus and one at the apex). The software automatically creates a line between the blood-tissue interface, which can be adjusted by the observer if the software cannot track accurately and calculates end-diastolic and end-systolic volumes for each view. When the software (“Auto-LVEF”) failed to correctly follow two or more ventricular segments per apical view were excluded from the analysis and considered unfeasible (Supplementary material; Appendix 4).

### Feasibility and reproducibility analysis

The echocardiograms and feasibility analysis were performed by Matías Pécora (MP), trained in Two-Dimensional Transthoracic Echocardiography, Doppler, and speckle tracking. For reproducibility analysis, 20 patients with feasible SL-S measurements were randomly selected. Intraobserver reproducibility was assessed by MP in two stages, separated by 4 weeks, without knowledge of the patient’s identity or previous results. Interobserver reproducibility was evaluated with observers who did not share the workstation or results. The second observer group included Piero Pastorini (PP) and Roberto Farolini (RF), both trained in echocardiography by MP [42]. Interobserver reproducibility was assessed by MP and PP for manual LVEF and manual SL-S measurements, and by MP and RF for automatic LVEF and automatic SL-S measurements.

### Statistical analysis

Normality tests (Kolmogorov–Smirnov) were performed on continuous variables. Depending on the distribution, parametric or non-parametric tests were applied. Continuous variables with normal distribution were expressed as mean and standard deviation (±1 SD); otherwise, they were expressed as median and interquartile range (IQR).

Dichotomous variables were presented as relative frequencies. Continuous variables were compared using Student’s *t* test or Mann–Whitney test as appropriate. Chi-square tests were used for dichotomous variables. Statistical analysis was conducted using SPSS version 19.

### Reproducibility by two methods

Intraclass Correlation Coefficient (ICC). Model: Two mixed forms; Type: Individual observer; Definition: Absolute agreement [43]. ICC values below 0.5 indicated poor reproducibility, 0.5–0.75 indicated intermediate reproducibility, 0.75–0.90 indicated good reproducibility, and above 0.90 indicated excellent reproducibility [30, 43]. ICC values were estimated with a 95% confidence interval (CI), and a *p* value <0.05 was considered statistically significant.

Bland & Altman analysis (BA). BA used the mean of the differences (average of the differences between observers), standard deviation (SD), and 95% limits of agreement (1.96 × SD ± mean difference) [30]. The decision to use both methods was made to complement the strengths and weaknesses of each of them. The ICC gives a dimensionless value of agreement, and although it does not have a strict cutoff point for adequate agreement, there are globally accepted margins. The ICC has limited value when comparing results in different populations. BA offers absolute reproducibility values, which can be plotted for better analysis and comparison, though it lacks universally accepted limits of agreement, requiring clinical judgment [30].

## Results

### Patient characteristics

Table 1 describes the general characteristics of the patients included (*n* = 21) and those excluded (*n* = 9). Among the included patients, ICU admission criteria were: 38% (*n* = 8) septic shock; 29% (*n* = 6) severe polytrauma; 9.5% (*n* = 2) cardiac arrest; 9.5% (*n* = 2) acute heart failure; 4.7% (*n* = 1) acute asthma; 4.7% (*n* = 1) postoperative neurosurgical; and 4.7% (*n* = 1) stroke. Among the excluded patients, ICU admission criteria were: 33% (*n* = 3) stroke; 22% (*n* = 2) traumatic brain injury; 11% (*n* = 1) Guillain–Barré syndrome; 11% (*n* = 1) exacerbation of chronic obstructive pulmonary disease; 11% (*n* = 1) severe polytrauma, and 11% (*n* = 1) community-acquired pneumonia. None of the excluded patients were admitted for sepsis or septic shock (*p* < 0.05 vs. included). No other significant differences were observed in the studied variables between the included and excluded groups. Conventional echocardiographic and SL-S parameters for the studied population are presented in Table 1.

**Table 1:** General and echocardiographic characteristics of the population

	Included (*n* = 21)	Excluded (*n* = 9)	*p* value
Age	53.14 (SD ± 16.38)	53.56 (SD ± 20.18)	0.95
Women *n* (%)	8 (38%)	2 (22%)	0.40
Obesity *n* (%)	4 (19%)	4 (44%)	0.14
Hypertension *n* (%)	9 (43%)	4 (44%)	0.94
COPD *n* (%)	6 (29%)	2 (22%)	0.71
Diabetes *n* (%)	5 (24%)	3 (33%)	0.59
CHF *n* (%)	3 (14%)	1 (11%)	0.82
AMI *n* (%)	4 (19%)	0 (0%)	0.16
CKD *n* (%)	0 (0%)	1 (11%)	0.12
SAPS 3	65.71 (SD ± 16.46)	61.00 (SD ± 11.24)	0.21
ICU mortality *n* (%)	9 (43%)	3 (33%)	0.62
ICU length of stay (days)	12.00 (IQR 6.50; 19.00)	25.00 (IQR 8.00; 32.50)	0.25
IMV days	10.00 (IQR 5.50; 18.00)	20.00 (IQR 5.50; 30.50)	0.28
IMV *n* (%)	21 (100%)	9 (100%)	0.98
PEEP (cmH_2_O)	8.50 (SD ± 3.01)	6.20 (SD ± 1.30)	0.05
Tidal volume (ml)	450.43 (SD ± 63.13)	492.2 (SD ± 52.60)	0.08
Noradrenaline *n* (%)	8 (38%)	3 (33%)	0.80
MAP (mmHg)	91.00 (SD ± 19.74)	99.40 (SD ± 13.30)	0.25
HR (beats/min)	87.43 (SD ± 15.71)	90.33 (SD ± 22.90)	0.73
Echo-time (days)	1.00 (IQR 0.50; 4.00)	4.00 (IQR 1.00; 9.00)	0.16
IVST (mm)	8.52 (SD ± 2.14)		
PWT (mm)	7.86 (SD ± 1.42)		
EDV (ml)	109.95 (SD ± 32.51)		
LVEF (%)	60.00 (IQR 53.00; 63.00)		
é average (m/s)	0.11 (SD ± 0.04)		
E/é	7.09 (IQR 6.13; 10.24)		
LAV (ml)	41.05 (SD ± 22.15)		
BDRV (cm)	37.50 (SD ± 6.36)		
TAPSE (mm)	21.60 (SD ± 6.07)		
Manual SL-S (%)	−18.50 (IQR −20.30; −13.85)		
Automatic SL-S (%)	−18.25 (IQR −20.48; −14.12)		
Manual SL-S-4C (%)	−18.30 (IQR −20.40; −14.50)		
Manual SL-S-2C (%)	−18.10 (IQR −21.35; −14.90)		
Manual SL-S-3C (%)	−18.30 (IQR −20.60; −12.40)		
Automatic SL-S-4C (%)	−17.10 (IQR −21.62; −13.10)		
Automatic SL-S-2C (%)	−17.45 (IQR −21.15; −16.12)		
Automatic SL-S-3C (%)	−17.45 (IQR −20.31; −15.20)		

Population characteristics: *n* (%), mean (± SD), or median (IQR) as appropriate

*COPD* chronic obstructive pulmonary disease, *CHF* chronic heart failure, *AMI* previous acute myocardial infarction, *SAPS 3* simplified acute physiology score III, *CKD* chronic kidney disease, *IMV* invasive mechanical ventilation, *PEEP* positive end-expiratory pressure, *MAP* mean arterial pressure, *HR* heart rate, *Echo-time* days after ICU admission to echocardiography evaluation, *IVST* interventricular septum thickness, *PWT* posterior wall thickness, *EDV* end-diastole volume, *LVEF* left ventricular ejection fraction, *LAV* left atrium volume, *BDRV* basal diameter of the right ventricle, *é average* diastolic tissue velocity average of the base of the LV septum and LV lateral wall, *E/é* E velocity pulse wave Doppler and é diastolic tissue velocity ratio, *TAPSE* tricuspid annular plane systolic excursion, *SL-S* LV global systolic longitudinal ventricular strain, *SL-S-4C* systolic longitudinal ventricular strain four-chamber view, *SL-S-2C* systolic longitudinal ventricular strain two-chamber view, *SL-S-3C* systolic longitudinal ventricular strain three-chamber view

### Feasibility

SL-S was feasible in 70% of cases (21 out of 30 patients). Among the nine excluded patients, six were due to an inadequate global view (two, three, four chamber), whereas three were due to an inadequate two chamber view, specifically for the inadequate visualization of the basal and medial segments of the anterior wall.

The feasibility of LVEF was 80% (24 out of 30 patients). Four participants were excluded due to an inadequate two-chamber view, and two due to an inadequate global view.

### SL-S: global and per-chamber reproducibility

**Fig. 1:**
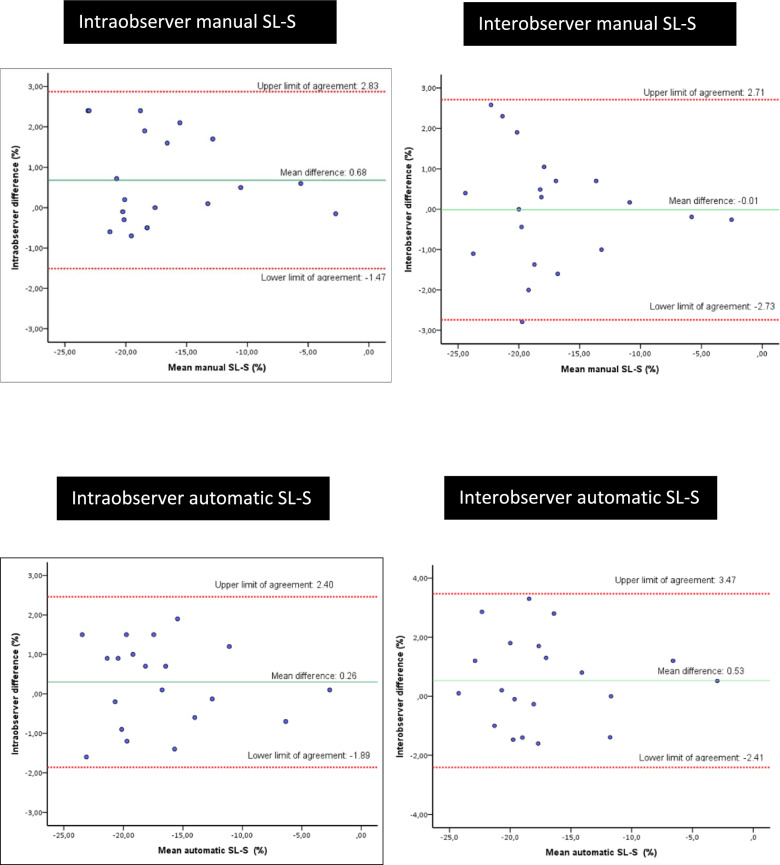
Bland–Altman graph illustrating the intra and interobserver reproducibility of global SL-S, mean measurements, difference, and 95% limits of agreement (1.96 SD) between observers

The reproducibility of SL-S, both globally and per chamber, for intraobserver and interobserver assessments, was excellent for both manual (Q analysis) and automatic (AFI) methods. ICC values are provided in Table 2, and BA values in Table 3. Figure 1 shows the BA plots illustrating intraobserver and interobserver reproducibility for SL-S using both automatic and manual methods.

**Table 2:** SL-S reproducibility: intraclass correlation coefficient (95% CI)

	Intraobserver reproducibility	Interobserver reproducibility
Manual SL-S	0.97 (0.91–0.99)*	0.97 (0.93–0.99)*
Automatic SL-S	0.97 (0.94–0.99)*	0.96 (0.92–0.98)*
Manual SL-S vs. automatic SL-S	0.97 (0.95–0.99)*	0.97 (0.93–0.99)*
Manual SL-S-4C	0.93 (0.67–0.98)*	0.93 (0.85–0.97)*
Manual SL-S-2C	0.91 (078–0.96)*	0.94 (0.87–0.97)*
Manual SL-S-3C	0.95 (0.88–0.98)*	0.91 (0.80–0.96)*
Automatic SL-S-4C	0.93 (0.83–0.97)*	0.93 (0.85–0.97)*
Automatic SL-S-2C	0.94 (0.85–0.98)*	0.92 (0.84–0.96)*
Automatic SL-S-3C	0.97 (0.92–0.99)*	0.93 (0.84–0.97)*
Manual LVEF	0.82 (0.48–0.93)*	0.78 (0.55–0.91)*
Auto-LVEF	0.94 (0.86–0.98)*	0.94 (0.87–0.97)*

Intra and interobserver reproducibility is represented by the intraclass correlation coefficient and 95% confidence interval (95% CI)

*SL-S* LV global systolic longitudinal ventricular strain, *SL-S-4C* systolic longitudinal ventricular strain four-chamber view, *SL-S-2C* systolic longitudinal ventricular strain two-chamber view, *SL-S-3C* systolic longitudinal ventricular strain three-chamber view, *Manual LVEF* left ventricular ejection fraction using the modified biplanar Simpson’s method, *Auto-LVEF* left ventricular ejection fraction using automatic GE^®^ software

*p < 0.001

**Table 3:** SL-S Bland–Altman reproducibility

	Intraobserver reproducibility	Interobserver reproducibility
Manual SL-S	0.68 (1.10); (−1.47 to 2.83)	−0.01 (1.39); (−2.73 to 2.71)
Automatic SL-S	0.26 (1.10); (−1.89 to 2.40)	0.53 (1.50); (−2.41 to 3.47)
Manual SL-S vs. Automatic SL-S	0.36 (1.09); (−1.77 to 2.49)	0.20 (1.20); (−2.15 to 2.55)
Manual SL-S-4C	1.42 (1.68); (−1.87 to 4.71)	−1.00 (1.90); (−4.72 to 2.72)
Manual SL-S-2C	0.18 (2.22); (−4.17 to 4.53)	−0.34 (2.01); (−4.28 to 3.60)
Manual SL-S-3C	0.64 (1.83); (−2.95 to 4.22)	1.10 (2.37); (−3.54 to 5.74)
Automatic SL-S-4C	0.82 (1.90); (−2.90 to 4.54)	1.10 (2.78); (−2.36 to 4.60)
Automatic SL-S-2C	0.24 (1.80); (−3.28 to 3.77)	−0.12 (2.14); (−4.00 to 4.00)
Automatic SL-S-3C	0.43 (2.16); (−3.80 to 4.66)	1.53 (1.80); (−1.99 to 5.00)
Manual LVEF	−5.00 (7.50); (−19.70 to 9.70)	7.50 (6.60); (−5.40 to 20.40)
Auto-LVEF	−0.95 (4.63); (−10.02 to 8.13)	1.75 (4.38); (−6.38 to 10.33)

Bland–Altman analysis represents intra- and interobserver reproducibility: the mean difference and its standard deviation (SD) are represented, as are the upper and lower limits of agreement (lower–upper)

*SL-S* LV global systolic longitudinal ventricular strain, *SL-S-4C* systolic longitudinal ventricular strain four-chamber view, *SL-S-2C* systolic longitudinal ventricular strain two-chamber view, *SL-S-3C* systolic longitudinal ventricular strain three-chamber view, *Manual LVEF* left ventricular ejection fraction using the modified biplanar Simpson’s method, *Auto-LVEF* left ventricular ejection fraction using automatic GE^®^ software

### LVEF reproducibility

Intraobserver and interobserver reproducibility for manual LVEF using Simpson’s method was good. In contrast, reproducibility for auto-LVEF was excellent. ICC values are provided in Table 2, and BA values are presented in Table 3.

## Discussion

To the best of our knowledge, this is the first study to exclusively evaluate the global and per-chamber feasibility and reproducibility of SL-S in ICU patients undergoing IMV.

The main results of the study are:

1. SL-S and LVEF demonstrated adequate feasibility in patients under IMV.
2. The intraobserver and interobserver reproducibility of global and per-chamber SL-S, using both manual and automatic methods, was excellent.
3. LVEF reproducibility ranged from good to excellent, with automatic measurement (auto-LVEF) outperforming manual measurement (Simpson’s biplane method).
4. SL-S reproducibility, whether manual or automatic, was greater than manual LVEF measurement and slightly higher than auto-LVEF.

### SL-S feasibility and reproducibility

SL-S is feasible in ICU patients undergoing IMV. The primary challenge lies in obtaining clear images, particularly in the two-chamber apical view, due to its anatomical relationship with the lung. This issue was reflected in our results, since one-third of excluded patients were due to inadequate imaging in the two-chamber apical view. In the included population, there was a significantly higher proportion of patients with septic shock. Whether septic shock enhances image quality for SL-S is uncertain. Studies in non-critically ill patients report feasibility ranging from 75 to 93% [10, 44]. Table 4 summarizes the findings on feasibility and reproducibility from international literature in the ICU population. Estimating SL-S in the four-chamber view could potentially enhance feasibility in the ICU. Bazalgette et al. [26] addressed this issue in a septic shock population. They evaluated the agreement between global SL-S and four chamber view with satisfactory results. This approach may simplify the use of SL-S, though further studies are needed to confirm these findings.

**Table 4:** Comparison of feasibility and reproducibility of GLOBAL SL-S in ICU

	Current study	Boissier et al. [15]	Nafati et al. [29]	Orde et al. [17]	Chang et al. [21]	De Geer et al. [31]	Yan et al. [45]
Population	(*n* = 30) 100% IMV SL-S	(*n* = 132) 100% Septic shock 80% IMV SL-S 2 & 4 chambers	(*n* = 64) 30% septic shock 39% IMV SL-S 4 chamber	(*n* = 60) 100% Sepsis/septic shock 65% IMV SL-S	(*n* = 111) 100% Sepsis/septic shock 65% IMV SL-S	(*n* = 47) 100% Septic shock 74% IMV SL-S	(*n* = 59) 100% Sepsis/septic shock^#^
Feasibility	SL-S 70% LVEF 80%	SL-S 44% LVEF 97%	SL-S 77% LVEF 84%	SL-S 81%	SL-S 93%	SL-S 94% LVEF 94%	SL-S 90%
*Reproducibility*							
Population	*n* = 20 100% IMV	X	X	*n* = 6^#^	*n* = 20^#^	*n* = 10 and 44 74% IVM	*n* = 30^#^
Intraobserver BA	Manual 0.68 (1.10); (−1.47 to 2.83) Automatic 0.26 (1.10); (−1.89 to 2.40)	X	X	−0.80 (0.50); (−1.78 to 0.18)	−0.71 (0.28); (−2.55 to 2.35)	X	−0.60 (0.94); (−2.5 to 1.2)
Interobserver BA	Manual −0.01 (1.39); (−2.73 to 2.71) Automatic 0.53 (1.50); (−2.41 to 3.47)	X	X	−0.90 (0.90); (−2.66 to 0.86)	−0.73 (0.28); (−1.96 to 3.03)	0.04 (2.60); (−5.00 to 5.10)	−1.10 (1.60); (−4.2 to 2.0)
ICC Intraobserver	Manual 0.97 (0.91–0.99) Automatic 0.97 (0.94–0.99)	X	X	X	0.88 (0.64–0.92)	0.89 (0.55–0.97)	0.97 (0.89–0.99)
ICC Interobserver	Manual 0.97 (0.93–0.99) Automatic 0.96 (0.92–0.98)	X	X	X	0.94 (0.88–0.98)	0.91 (0.74–0.95)	0.91 (0.73–0.97)

Reproducibility is represented by Bland–Altman analysis (BA): the mean difference and its standard deviation (SD) are defined by the upper and lower limits of agreement (lower–upper)

Reproducibility is represented by the Intraclass Correlation Coefficient (ICC) and its 95% confidence interval (95% CI)

*IMV* Invasive mechanical ventilation, *SL-S* global systolic longitudinal ventricular strain

X: not reported in the cited study

^#^It does not describe the percentage of IMV or general characteristics

The intraobserver and interobserver reproducibility of global and per-chamber SL-S, using both manual and automatic methods, was excellent. Four reports of global SL-S reproducibility [17, 21, 31, 45] are summarized in Table 4. To date, there are no reports on per-chamber SL-S reproducibility in the ICU. Our results showed slightly lower reproducibility compared to non-critically ill populations, though the levels remain excellent and consistent with findings in critically ill patients, including those under IMV [10]. When considering our results in ICU population, the levels are comparable, even when only including patients under IMV. Notably, the excellent reproducibility of SL-S in the four-chamber view suggests that focusing on this view alone could improve feasibility, particularly for assessing global LV dysfunction such as septic cardiomyopathy [26]. SL-S could be a reliable assessment tool in the ICU, but additional studies are needed to validate these findings due to the limited number of reproducibility reports.

### LVEF feasibility and reproducibility

Comparing our results with existing literature, the feasibility of LVEF in our study was slightly lower than the reported range of 84–93% [29, 31]. This difference may be due to our study’s inclusion criteria of 100% of patients under IMV. LVEF estimations based on biplane Simpson’s method of discs (manual LVEF) suffer from modest reproducibility. As a rule of thumb, LVEF changes of less than 10% points between examinations or examinator do not necessarily represent an actual change in systolic function [9, 46, 47]. However, reproducibility studies of LVEF in non-critical patients report great variability results [10, 48–50]. Our results are within the range reported in non-critical care settings, though slightly lower reproducibility for manual LVEF measurement and higher reproducibility for auto-LVEF. Variations in LVEF reproducibility may be attributed to differences in image quality. The automatic method includes post-processing that enhances the visibility of the blood-tissue interface, providing a clearer definition of endocardial borders. Additionally, automated software aims to minimize observer variability and achieve greater standardization [48]. When comparing the ICU population, our results fall within the reported ranges (even in patients under IMV only), though slightly lower reproducibility for manual LVEF measurement and higher reproducibility for auto-LVEF, suggesting that automatic measurement can improve LVEF reproducibility in the ICU context [31, 32, 51]. Manual measurements are time-consuming and operator dependent. Automated algorithms have been developed to facilitate the measurement of LVEF and reduce observer variability [52]. In the context of the ICU, Varudo et al. [53] evaluated an AI-based tool to automate LVEF measurement in a single four-chamber apical view in 95 critically ill patients (34% of them under invasive mechanical ventilation). A reproducibility analysis of automated LVEF measurements was performed across two distinct groups of echocardiographers, namely novices and experts. Manual LVEF measurements using the biplane Simpson’s method, performed by an expert, were considered the reference standard. Interobserver reproducibility of the automated method was comparable to that reported in our study. Moreover, intraobserver reproducibility was significantly better with the automated technique compared to the manual reference method ( Simpson’s biplane). Although further studies are warranted to confirm these findings, the validation and implementation of automated software in the critical care setting could enhance the reproducibility of LVEF assessment. Ultimately, this could enhance the potential clinical utility of automated LVEF measurements in critically ill patients.

### SL-S reproducibility versus LVEF reproducibility

We evaluated the variability of SL-S in comparison to LVEF, as LVEF is the most assessed parameter for left ventricular systolic function in the ICU setting. SL-S (automatic and manual) showed greater reproducibility than manual LVEF and was slightly better or comparable to auto-LVEF. These results align with existing literature for both non-critical and ICU populations [10, 31].

### Limitations

This study has several limitations that should be acknowledged. (1) The main limitation is the relatively small sample size and the monocentric design, which may affect the statistical power and generalizability of the findings. (2) We did not exclusively include patients with sepsis and/or septic shock. However, the studied population consisted of critically ill patients, characterized by high SAPS 3 scores, elevated mortality rates, prolonged ICU stays, and prolonged mechanical ventilation. (3) It is unclear whether the higher proportion of patients with septic shock has led to a greater feasibility of strain analysis. Among the variables described as limiting in the ICU for an adequate echocardiographic window (IVM, obesity, COPD, tidal volume, and PEEP), no differences were observed between the included and excluded populations. (4) Other systolic function parameters—such as MAPSE, Mitral Valve E-Point to Septal Separation, and longitudinal systolic velocity measured by Doppler tissue imaging—were not included for comparison with SL-S feasibility and reproducibility. These alternative measures deserve to be explored in future studies. (5) Despite randomization, most patients in our study had preserved ejection fraction and normal LV dimensions. This may restrict the applicability of our findings to patients with reduced LVEF and dilated LV. Future research could address this gap.

## Conclusion

The assessment of SL-S, both globally and per chamber, as well as auto-LVEF are feasible and showed excellent reproducibility in ICU patients under IMV. SL-S is the most reproducible parameter. Therefore, SL-S and Auto-LVEF may thus represent a useful tool in the evaluation of LV function in ICU patients under IMV.

## Supplementary Information

**Additional file 1.**

## Data Availability

The datasets used and/or analysed during the current study are available from the corresponding author on reasonable request.
