# Supplementary material for: Left ventricular systolic longitudinal strain in mechanically ventilated patients in the intensive care unit: assessment of global and chamber reproducibility
Source: Intensive Care Med Exp. 2025 Jun 17;13:62. doi: 10.1186/s40635-025-00770-8 (PMC12173981; doi:10.1186/s40635-025-00770-8)
Supplement: Supplementary file 1 — Additional file 1. [file 40635_2025_770_MOESM1_ESM.docx]

# SUPPLEMENTARY MATERIAL

## Appendix 1: Patient selection criteria in a step-by-step process

Feasibility of SL-S and LVEF was evaluated


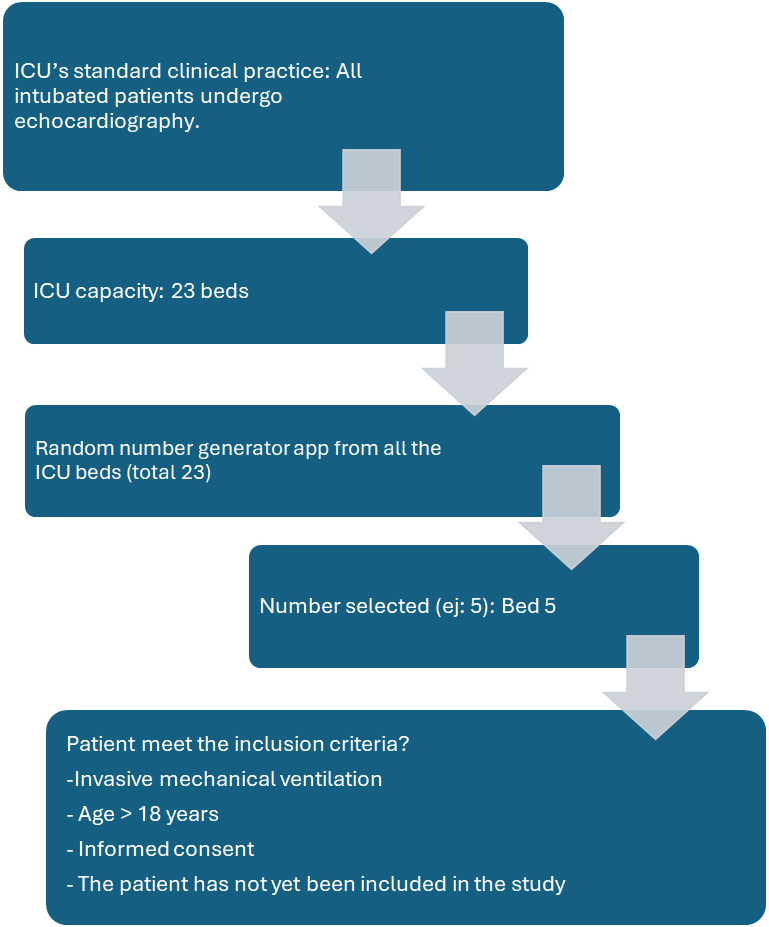

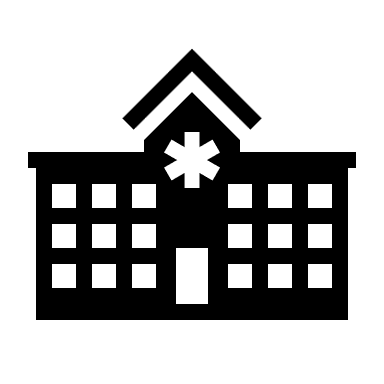

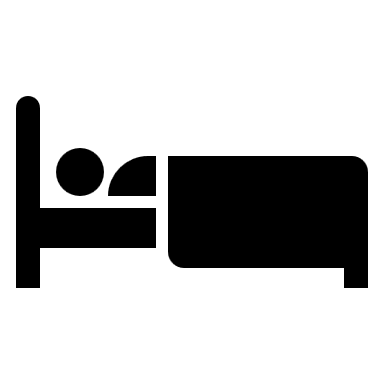

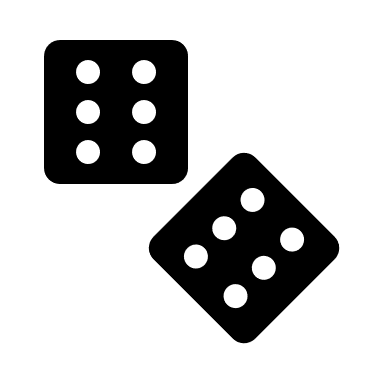

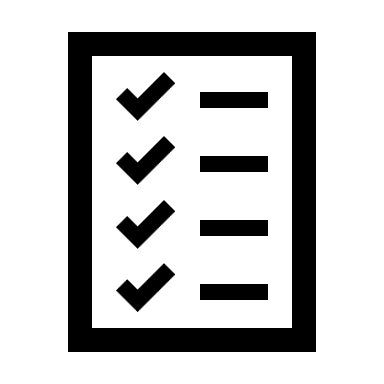

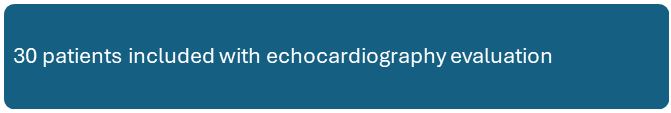


Intraobserver and interobserver reproducibility of SL-S and LVEF in 20 patients

## Appendix 2: SL-S acquisition by "Automated Functional Imaging" (AFI).

Figure 1A: Four chamber view

Figure 1B: Two chamber view

Figure 1C: Three chamber view


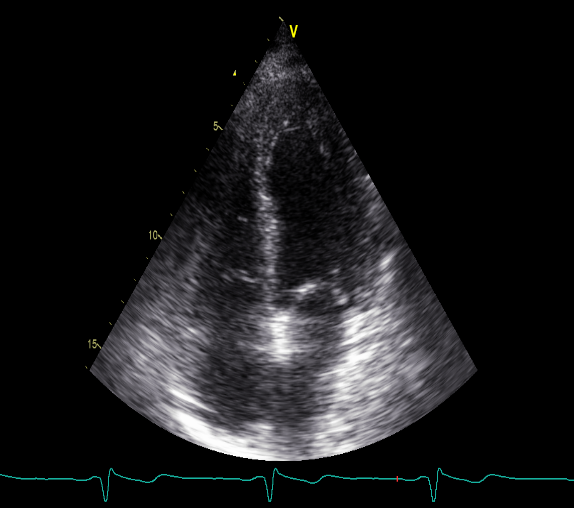

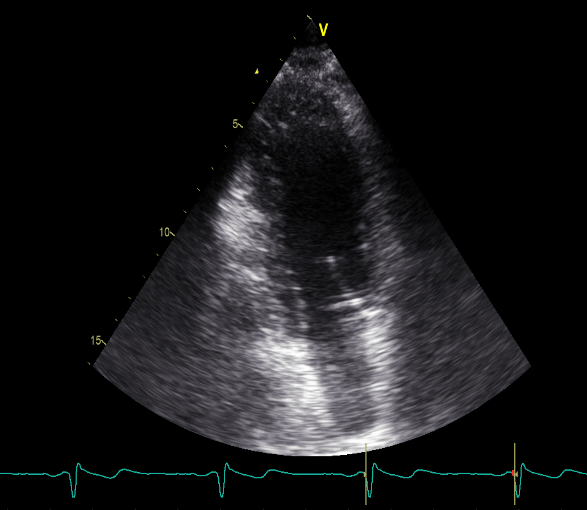

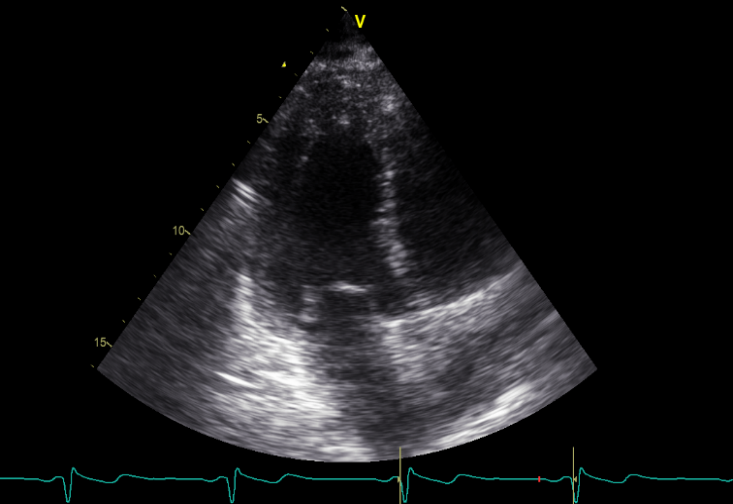


This method involves the operator defining three points (yellow dots) for each apical view. In the four-chamber view (Figure 1A), the points are placed at the junction of the mitral annulus with the lateral wall, the junction of the mitral annulus with the interventricular septum, and at the level of the apex. In the two chambers view (Figure 1B), the points are placed at the junction of the mitral annulus with the anterior wall, the junction of the mitral annulus with the inferior wall, and at the level of the apex. In the three chambers view (Figure 1C), the points are placed at the interventricular septum (excluding the LV outflow tract), at the junction of the mitral annulus with the inferolateral wall, and at the level of the apex.

Figure 2B; Four-chamber view: incorrect follow-up assessment by AFI

Figure 2A; Four-chamber view: Correct follow-up assessment by AFI.


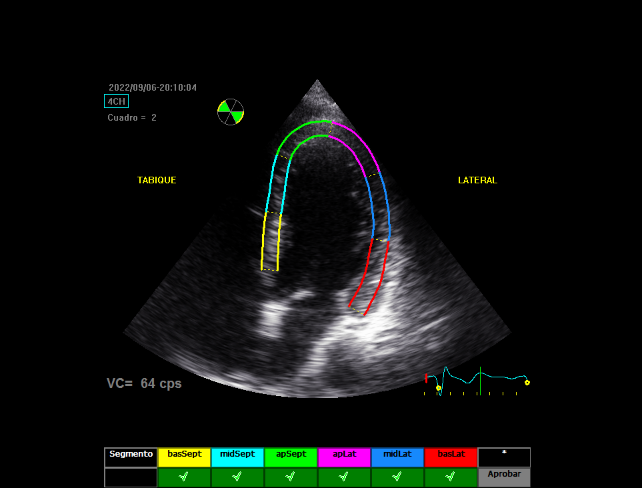

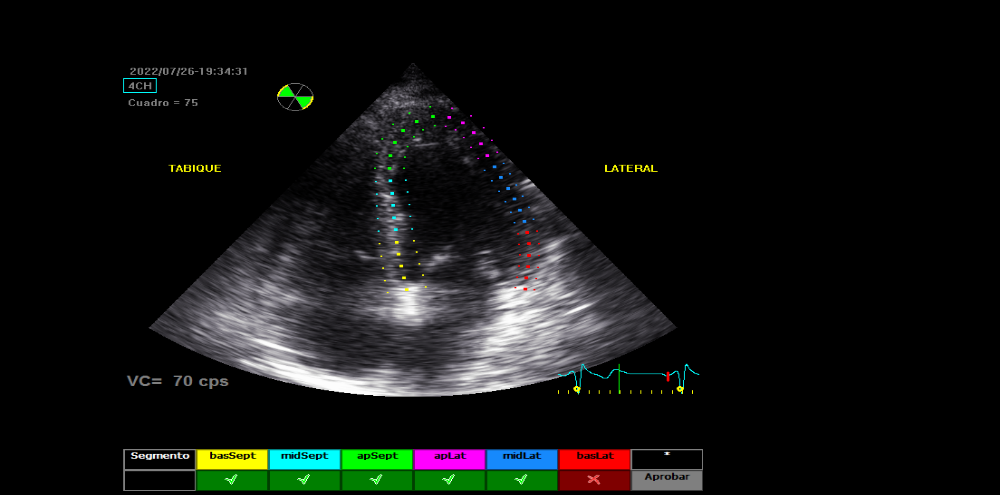


After defining the three points of interest for each view, the software displays the region of interest on the ventricular wall corresponding to each view (Figure 2A; four chamber view).

Each apical view is divided into six equal segments, and the software indicates the quality of tracking for each segment with either a tick (✔) or a cross (✘). If tracking is unsuccessful (indicated by a cross; Figure 2B), the operator can manually adjust the region of interest using anchor points on the ventricular wall and reassess whether the software can adequately track the segment. If the software still fails to track the segment properly (again indicated by a cross), that segment is excluded from the ventricular strain analysis.

The software's inability to adequately track two or more ventricular segments per view was defined as a non-feasibility criterion for strain analysis.

Figure 3B: Bull's eye and global SL-S

Figure 3A: SL-S four-chamber view


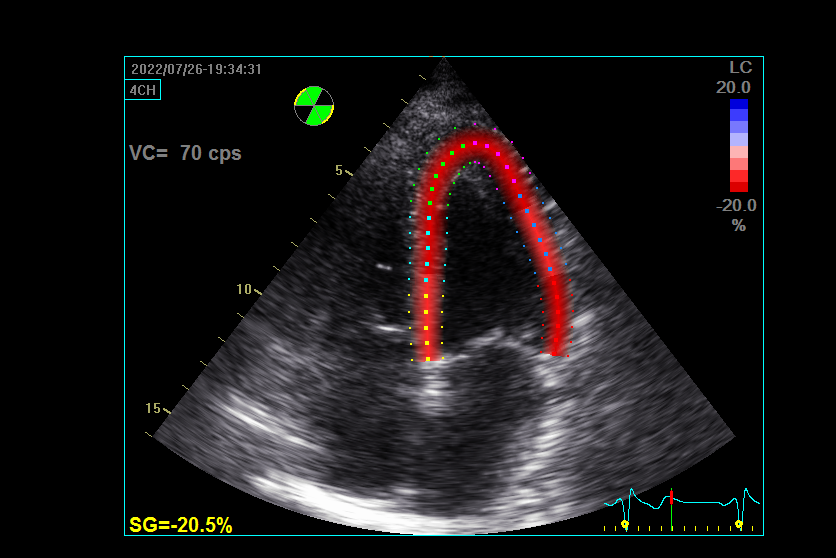

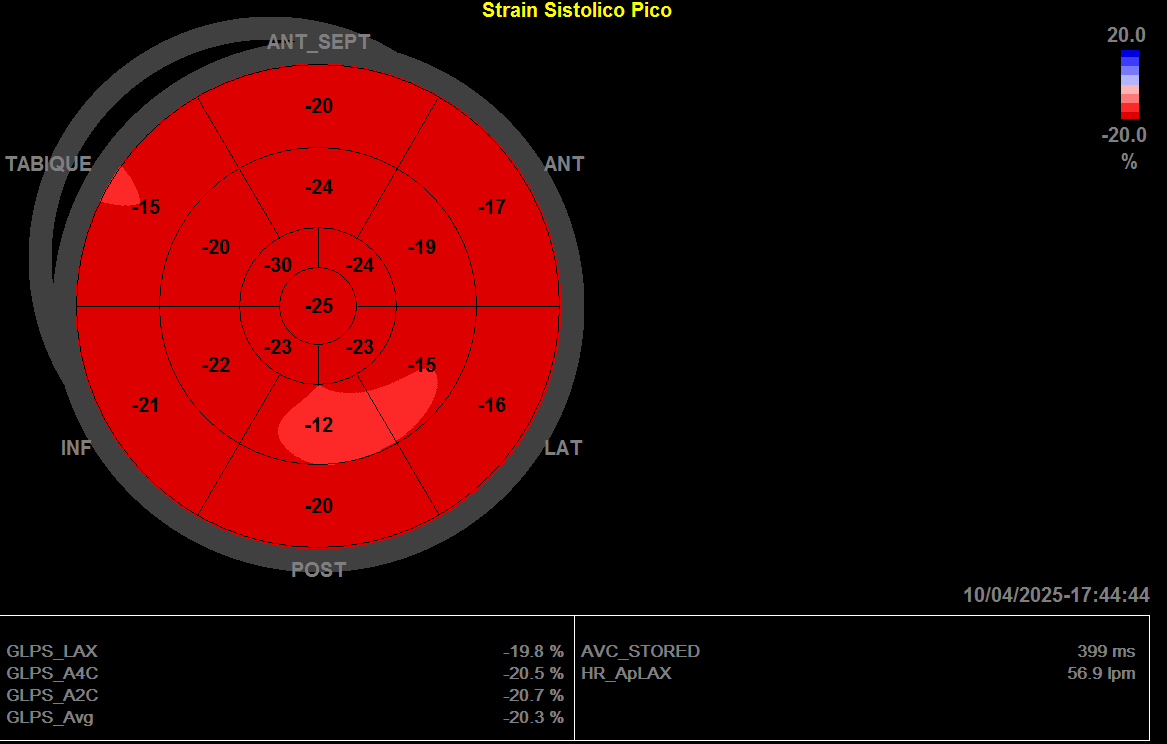


The peak value of SL-S is provided for each apical view such as the four chamber view (Figure 3A; yellow box). Figure 3B displays the bull's eye with the peak SL-S values for each ventricular segment. The yellow box highlights both the peak SL-S values for each view and the average value. (GLPS_LAX: global longitudinal peak systolic three chamber view; GLPS_A4C: global longitudinal peak systolic four chamber view; GLPS_A2C: global longitudinal peak systolic two chamber view; GLPS_Avg: global longitudinal peak systolic average value of SL-S).

## Appendix 3: SL-S acquisition by "Q analysis”.

Figure 4: SL-S four-chamber view Q analysis


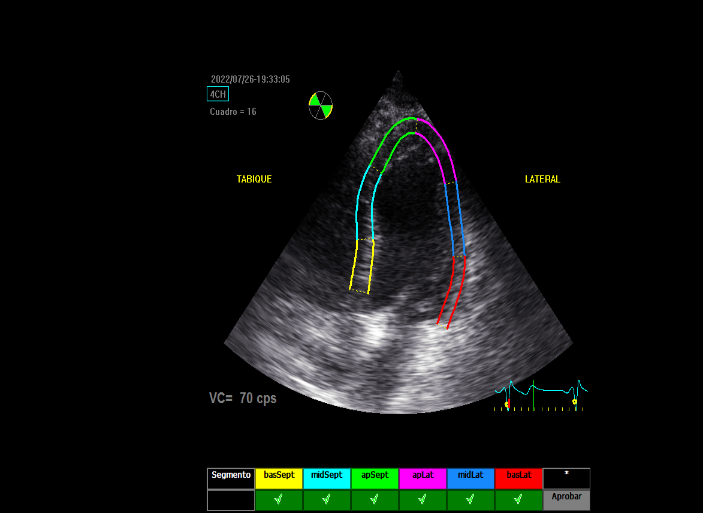

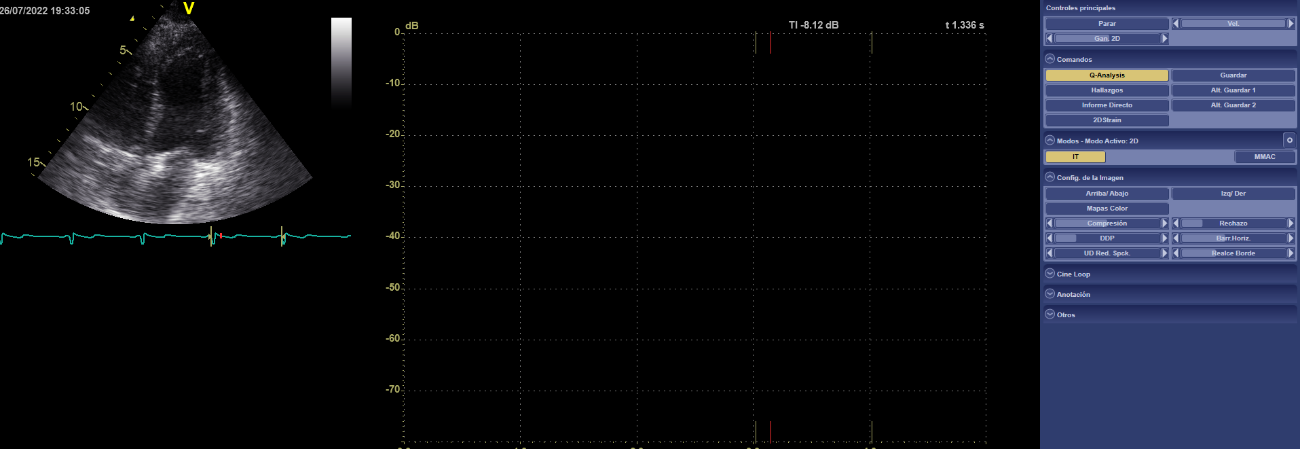


The endocardial borders of the left ventricle were manually traced in the apical two, three, and four chamber views. The width of the region of interest was adjusted according to the thickness of the ventricular wall, encompassing both the endocardial and epicardial borders. The software then divided the ventricular wall into six equal segments.

Figure 5B: Strain tracing for each ventricular segment during the cardiac cycle.

Figure 5A: Tracking Q analysis four chamber view


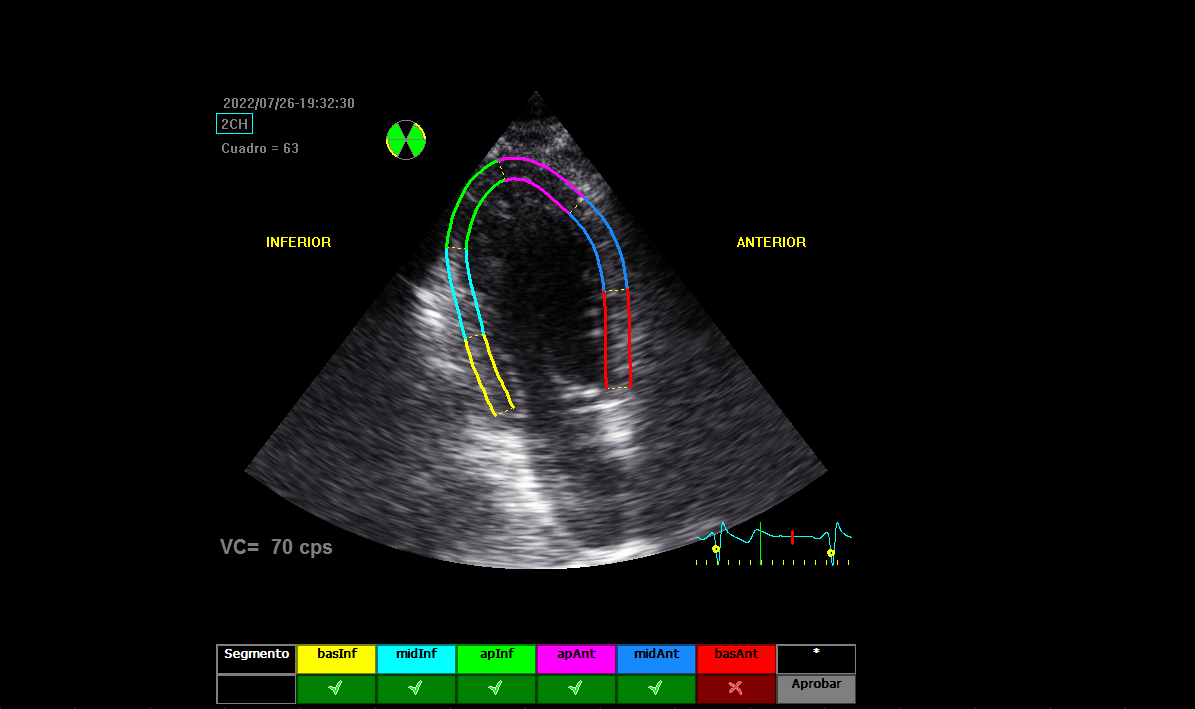

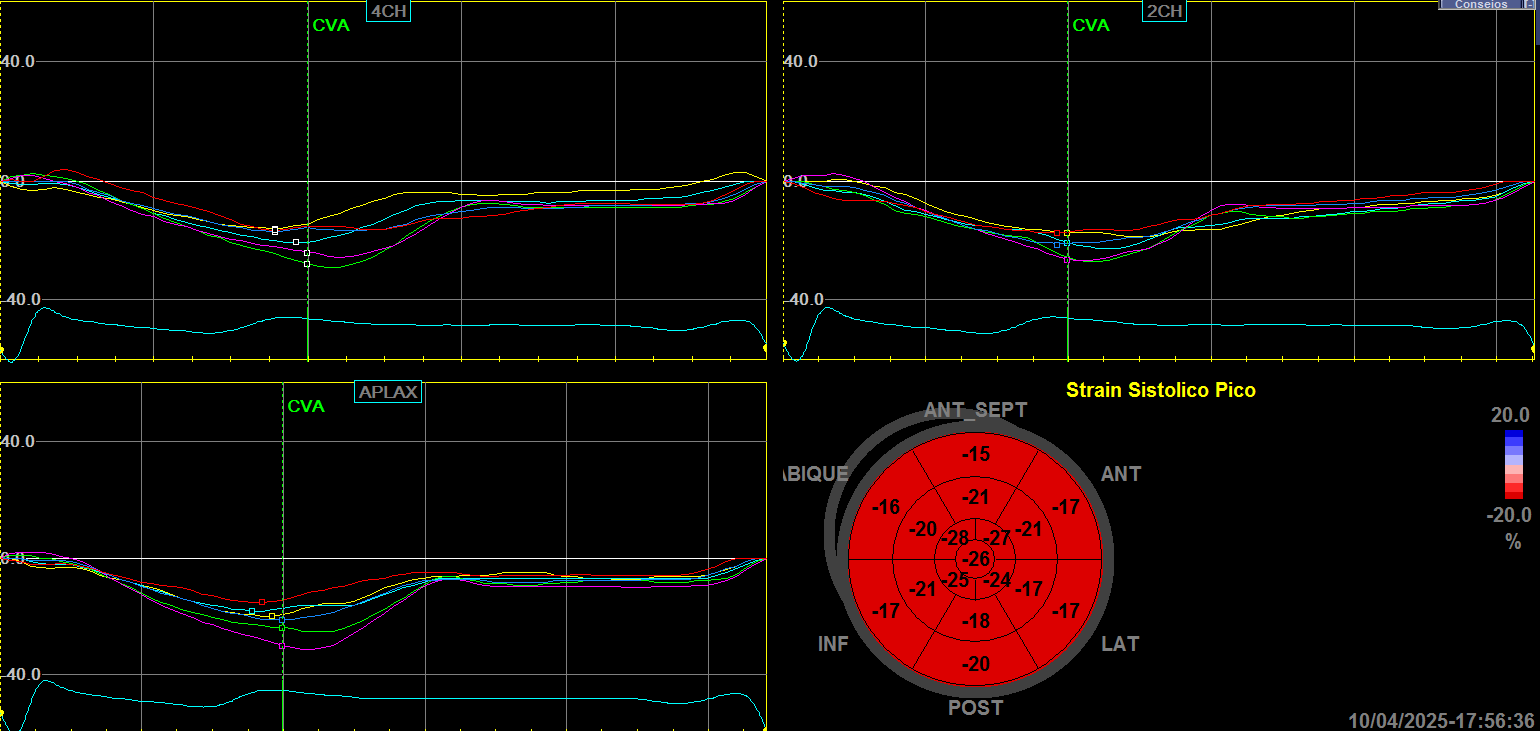


Before generating the results, the observer assessed the tracking quality of each segment by monitoring the region of interest (ROI) represented by colored rectangles on the ventricular walls throughout the cardiac cycle (Figure 5A) and the corresponding strain tracings (Figure 5B). The observer could manually adjust the ROI if necessary. Segments deemed to have inadequate tracking were excluded from the ventricular strain analysis. Inadequate tracking, defined by the observer, of two or more ventricular segments per view was considered a criterion for the non-feasibility of strain analysis. Figure 5B: The colored unfilled squares represent the peak strain value of each of the six ventricular segments. CVA: Aortic valve closure.

Figure 6: Peak values of SL-S per chamber and Bull's-eye from Q analysis


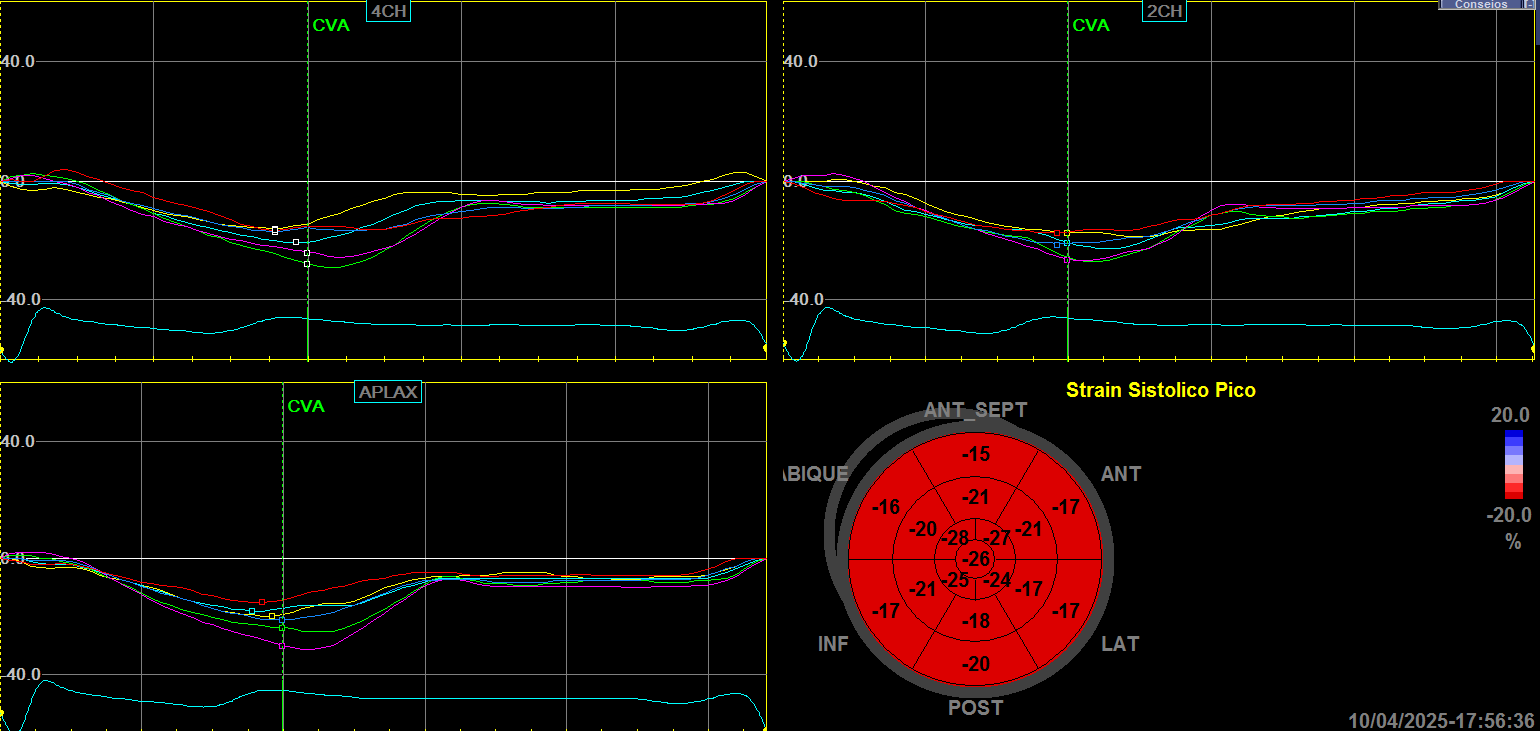


Finally, the peak SL-S values are obtained for each apical view (APLAX: three chamber view; 4CH: four chamber view; 2CH: two chamber view) and are displayed in a bull’s-eye plot (Figure 6). CVA: Aortic valve closure. The unfilled squares represent the peak strain value of each of the six ventricular segments, for each of the views.

Figure 7: Bull's eye and global SL-S by Q analysis


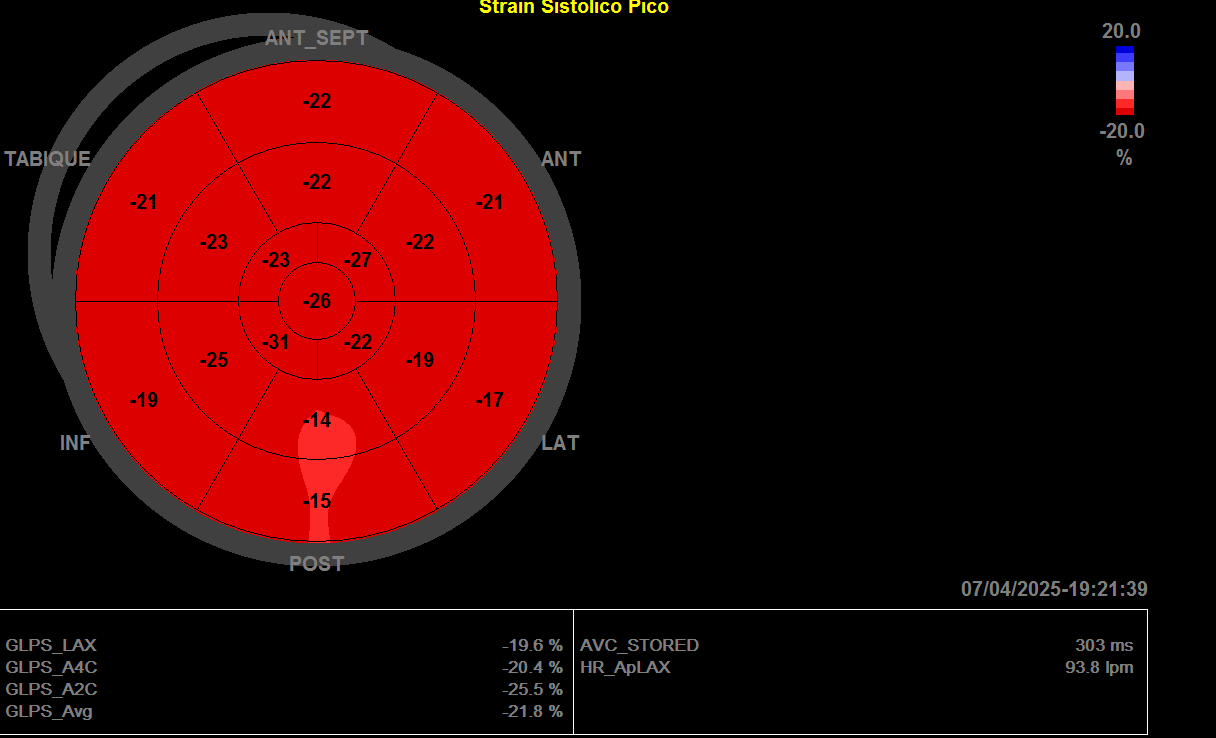


Figure 7 shows the bull’s-eye plot illustrating the peak SL-S values per ventricular segment, with the yellow box indicating both individual apical view values and the global average. (GLPS_LAX: global longitudinal peak systolic three chamber view; GLPS_A4C: global longitudinal peak systolic four chamber view; GLPS_A2C: global longitudinal peak systolic two chamber view; GLPS_Avg: global longitudinal peak systolic average of SL-S).

## Appendix 4: LVEF acquisition by "Auto LVEF”.

Figure 7A: Auto-LVEF four chamber view


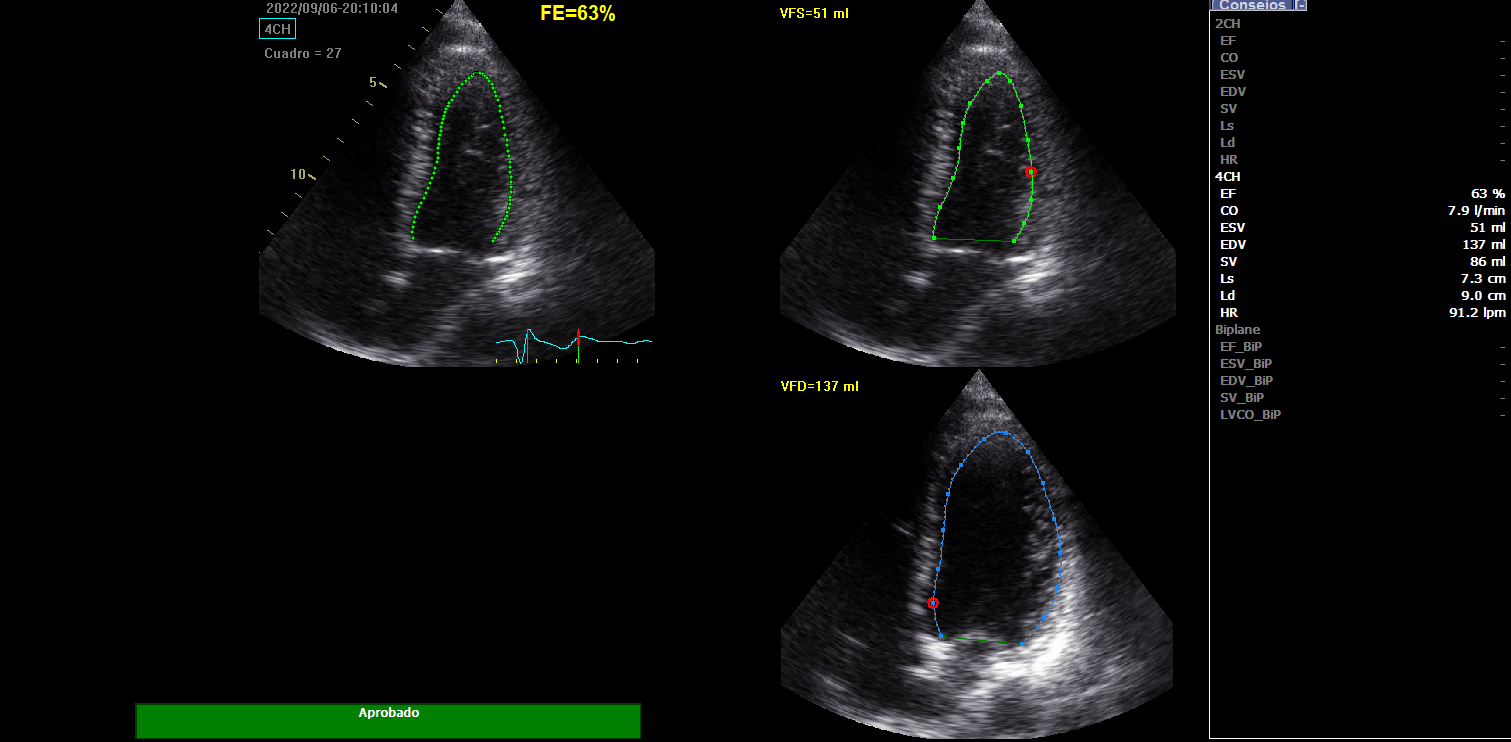


Figure 7B: Auto-LVEF two chamber view


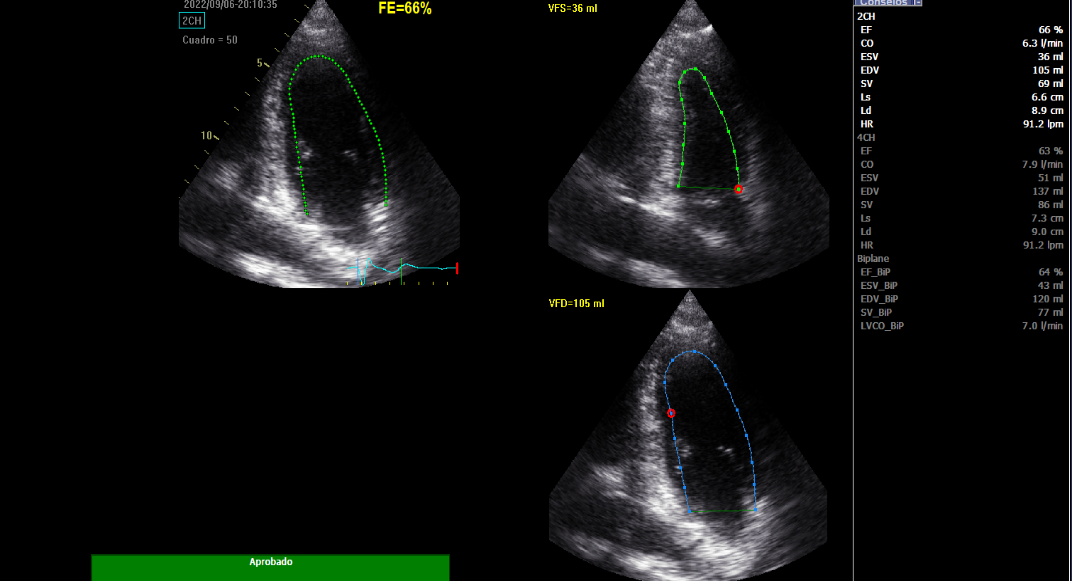


The Auto LVEF measurement involves defining three points in both the four-chamber and two-chamber views. In the four chamber view (Figure 7A), the points are placed: 1- at the junction of the interventricular septum and the mitral annulus; 2- at the junction of the lateral wall and the mitral annulus; 3- at the level of the apex. In the two chamber view (Figure 7B), the points are placed: 1- at the junction of the anterior wall and the mitral annulus; 2- at the junction of the inferior wall and the mitral annulus; 3- at the level of the apex. The software then automatically traces the blood–tissue interface in each view. If the software fails to accurately follow any part of the endocardial border (highlighted in red), the observer can manually adjust the tracing until it is correct. Subsequently, the software records the end-diastolic and end-systolic volumes, as well as the LVEF, for each view, along with a frozen frame and the corresponding blood–tissue interface tracing at that phase of the cardiac cycle.

Apical views with inadequate tracking of two or more ventricular segments per view by the Auto-LVEF software were excluded from the analysis, even after operator adjustments (e.g., persistent tracking failure in the basal and mid segments of the anterior wall).
